# Toward In Silico NAMs Analysis of Thyroid Disruption Leading to Developmental Neurotoxicity—A Collection of AOP-Anchored Computational Models

**DOI:** 10.1021/acs.chemrestox.5c00326

**Published:** 2026-03-31

**Authors:** Beata Judzinska, Wiktor Nisterenko, Natalia Bulawska, Dominika Kowalska, Karolina Jagiello, Anita Sosnowska, Tomasz Puzyn

**Affiliations:** † Laboratory of Environmental Chemoinformatics, Faculty of Chemistry, University of Gdańsk, Wita Stwosza St. 63, Gdańsk 80-308, Poland; ‡ Department of Physical Chemistry, Medical University of Gdańsk, Al. Gen. Hallera 107, Gdańsk 80-416, Poland; § QSAR Lab Ltd., Grunwaldzka Ave. 103A, Gdańsk 80-244, Poland

## Abstract

Thyroid disruption (TD) plays a critical role in developmental neurotoxicity (DNT), given the essential functions of thyroid hormones in brain development. The identification and assessment of DNT caused by TD have become a significant focus in regulatory toxicology, necessitating the use of innovative approaches that are both predictive and efficient. This study provides a comprehensive examination of in silico new approach methodologies, with a particular emphasis on (quantitative) structure–activity relationship ((Q)­SAR) models. Models anchored in the adverse outcome pathway framework offer mechanistic insights and predictive capabilities for assessing DNT linked to TD. By integrating knowledge of molecular initiating events and key events associated with thyroid hormone disruption, quantitative structure–activity relationships models provide a streamlined approach for predicting DNT. This systematic review identified 44 relevant studies documenting a total of 178 predictive models. The distribution of models across endpoints reveals that the most dominant endpoints are PXR (72), TTR (45), and TPO (21). A rigorous quality assessment showed that only 32 models are fully compliant with the OECD QAF (Quality Assessment Framework) criteria. This highlights the urgent need for more robust, endpoint-specific modeling tools.

## Graphical abstract

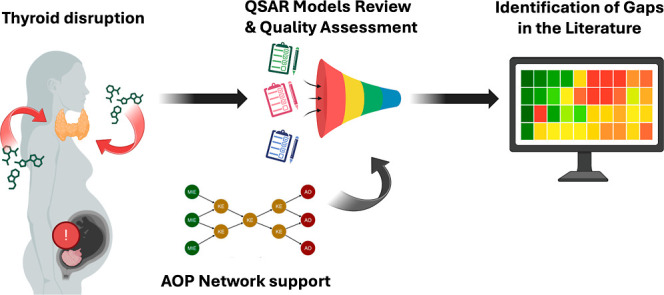

## Introduction

The toxic impact of chemicals on the nervous system is profoundly influenced by the maturity of the organism at the time of exposure to a stressor. It can be distinguished into developmental neurotoxicity (DNT) and adult neurotoxicity. The diverse effects of exposure in adults and children are not only due to differences in nervous system maturity but also due to reduced detoxification capacity in young ones, in whom the full development of defense mechanisms demands time.^1^

Although DNT is increasingly recognized as a major public health concern, regulatory frameworks continue to face challenges in establishing protective reference values for many chemicals, particularly under low-dose and chronic exposure scenarios. These limitations stem largely from conventional in vivo testing approaches, which are resource-intensive, time-consuming, and often insufficiently sensitive to detect subtle neurodevelopmental alterations. Consequently, many chemicals currently in use lack comprehensive DNT hazard characterization, especially during critical windows of brain development.

Regardless of the undeniable importance of addressing neurotoxic exposure, arising from the fact that disturbing any stage of brain development might have long-term consequences for the further functioning of an adult individual, progress in this field remains slow. The researchers Grandjean and Landrigan in 2006, referred to this ongoing issue as the “silent pandemic” of neurodevelopmental disorders. The difficulty in identifying this effect arises from the absence of clinically visible symptoms of toxicity, particularly in the context of chronic exposure to low doses, which complicates diagnosis and intervention. Furthermore, as mentioned above, a majority of existing chemicals, along with novel substances introduced to the market, remain untested for DNT. In effect, these potentially harmful chemicals continue to be released into the environment.^1^ Recent statistics show the alarming scale of this problem: only 110–140 out of approximately 350,000 compounds registered worldwide have been assessed for DNT. This situation is certainly attributed to the lack of mandatory DNT testing during the registration of new substances across regulatory sectors.^2^ Promising changes are on the horizon. The registration amendment is planned for Biocidal Products in the European Chemical Agency (ECHA). According to current legislation, DNT is located in the so-called additional data set, and this endpoint is assessed only if such a need is noticed based on the other findings. After implementing the adjustments in Annex II of the Biocidal Product Regulation (BPR), this endpoint will be shifted to the core data set; thereby, DNT testing will be obligatory.^3^

Currently, two directives govern the DNT assessment: OECD (Organization for Economic Co-operation and Development) test guideline (TG) 426 ^4^ and cohorts 2A and 2B of an Extended One-Generation Reproductive Toxicity study (OECD TG 443 ^5^) with an additional investigation for cognitive functions. Both are in vivo tests requiring at least 20 pregnant rats per dose group across three different dose levels and a control group.

Although the protocols specify that these studies typically span 70 to 90 days, a report by the ECHA titled “Analysis of capacities and capabilities of laboratories to conduct OECD TG 443 extended one-generation reproductive toxicity study” highlights a different reality. The report states that the average time from signing a contract with a laboratory to obtaining results for the DNT assessment is approximately 15 months. This extended time frame is primarily due to factors not directly associated with the experiment duration but rather are the consequences of the availability of animal facilities, dose selection, and breeding of animal strains. Thus, current DNT assessment studies are complicated, time-consuming, and expensive and use a sizable number of animals.^6^

Despite the challenges associated with the DNT assessment method, no alternatives with better parameters are currently accepted in any registration process for new chemicals. This has driven the necessity of developing New Approach Methodologies (NAMs) that refer to alternative test methods and strategies to reduce, refine, or replace (3R) vertebrate animals.^7^ NAMs encompass in silico, in chemico, and in vitro approaches, along with innovative tools like high-throughput screening and high-content methods and thus hold great promise as a critical avenue for advancing the process of efficient DNT assessment.^8^

Recently, international efforts have been made to develop an in vitro battery (IVB) for DNT assessment. This approach aims to evaluate the effect of substances on various stages of neurodevelopmental processes such as proliferation, migration, differentiation, network formation and function, and apoptosis.^9^ IVB consists of ten individual NAMs that utilize human neural cells at different developmental stages, offering a more human-relevant model for testing compared to the traditional method. However, despite promising capabilities, the IVB approach has limitations. It does not yet take into account the several key mechanisms that are responsible for inducing the toxic neurodevelopmental effect, such as oxidative stress, neuroendocrine disruption, immune system disruption, or other indirect causes related to the liver, kidneys, pancreas, and gastrointestinal tract.^10^ These gaps underscore the need for further development of NAMs for comprehensive assessment of substances that may be linked to the risk of DNT to promptly protect children’s brains.

It has been established that thyroid disruption (TD) in pregnant women affects the development of the fetal nervous system.^11^ TD can increase the risk of epileptic seizures occurrence, autism spectrum disorders, and attention deficit hyperactivity disorder.^12^ A wide range of environmental contaminants are known to interfere with thyroid function. These include pesticides, perfluoroalkyl substances (PFAS) used as surface coatings for textile, food, and cosmetic packaging production, and also industrial chemicals, such as polychlorinated biphenyls (PCBs), polybrominated diphenyl ethers (PBDEs), perchlorate, bisphenol A (BPA), and phthalates. Alarmingly, even exposure to very low concentrations of these substances can lead to adverse effects (14). This highlights the urgent need to identify potential risks and establish preventive measures to minimize exposure. Currently, as well as for DNT, endocrine disruption is not obligatory in toxicity testing, whereas the revision of Annex II of the BPR will also promote this endpoint to the core data set.^3^ Although NAMs specifically dedicated to DNT remain limited, substantial progress has been made in developing in vitro and computational approaches addressing related mechanisms. Given the close association between DNT and endocrine disruption, particularly TD, NAMs designed for TD assessment may provide valuable insights and serve as a foundation for future DNT-focused methodologies. Harmonized TD-related guidelines could therefore play a crucial role in advancing DNT assessment frameworks.^9,13^

Building on the role of Adverse Outcome Pathways (AOPs) as structured frameworks linking Molecular Initiating Events (MIEs), Key Events (KEs), and Adverse Outcomes (AOs), their application in understanding TD-induced DNT is particularly significant. In the context of TD-DNT, AOPs enable the identification of measurable and biologically plausible endpoints crucial for developing NAMs. They present a sequence of causal relationships at different levels of biological organization, beginning with a MIE and leading to an AO with KEs. For example, binding to thiol/selenol groups in selenoproteins can result in structural modifications, inhibiting catalytic activity and impairing metabolic capacity to neutralize reactive oxygen species.^5,16,17^ The essential components of AOPs are KEs, which provide measurable changes in biological systems that can prioritize substances for toxicity testing, develop biomarkers for diagnostics, and advance targeted therapeutics that interrupt toxic pathways before harmful outcomes occur.

AOPs often share KEs (including MIEs and AOs), creating complex AOP networks (AONs) in real-world scenarios. Tools like AOP-Wiki capture, disseminate, and review AOP knowledge, providing detailed pathways, including prototypical stressors and taxonomic applicability.^14^ However, the availability of fully developed AOPs remains limited, underscoring the need for further development.

In this context, models anchored in AOPs, such as (Quantitative) Structure–Activity Relationship ((Q)­SAR) models, are invaluable for evaluating chemicals to determine their potential to cause specific events with the pathways, which are key contributors to neurodevelopmental impairments. These tools, when combined with AOP knowledge, provide a robust framework for addressing gaps in TD-DNT research while advancing the utility of NAMs.

Despite the challenges associated with in vivo testing and the growing obligation to assess DNT and endocrine disruption for biocides, there is an international focus on advancing alternative approaches. Methods such as quantitative structure–activity relationships (QSAR) models and read-across approaches are central to these recommendations.^15^ QSAR models allow for the assessment of chemicals, determining whether they are likely to trigger KE(s) and distinguishing between neurotoxic and non-neurotoxic compounds as part of an integrated computational prediction system. Additionally, high-quality QSAR models facilitate the rapid screening of large compound data sets. By providing information about potential MIEs and their associated mechanisms of toxicity, QSAR models help prioritize chemicals for more targeted screening or testing, such as in vitro or in vivo studies.

This publication aims to provide a comprehensive review of available in silico NAMs, particularly predictive or QSAR models, that are anchored in AOPs and applicable for assessing DNT triggered by TD. This review consolidates existing knowledge, evaluates the current state of available models, and identifies critical gaps that are specific to TD-DNT pathways. A systematic review, following the “Preferred Reporting Items for Systematic Reviews and Meta-Analyses” (PRISMA) methodology,^16^ was conducted to extract relevant literature on in silico models for KEs in the developed AON. Finally, the quality of these models was assessed according to OECD’s “Guidance Document on the Validation of (Quantitative) Structure–Activity Relationship [(Q)­SAR] models”. To achieve this, AOPs describing the impact of TD on DNT were reviewed, and a network of KEs, which decreased thyroid hormones, was constructed. This study summarizes the available models, evaluates their applicability for predicting TD events leading to DNT, and outlines the specific gaps in modeling tools for TD-DNT pathways. The review provides a rapid and accessible resource for identifying high-quality models for evaluating chemicals that can trigger specific KEs while facilitating feature research to address these gaps and enhance coverage of TD-DNT mechanisms.

## Methodology

### Methodology of the Development of AON for DNT Caused by TD

In this work, we reviewed the available AOPs in the AOP-Wiki that describe the DNT triggered by the TD. To avoid unnecessary replication of effort, the data summarized in the review article by Marty et al. in 2021 have been utilized.^17^ Furthermore, using the search terms “T4”, “thyroxine”, “T3”, “triiodothyronine”, and “thyroid”, we have also conducted an analysis of the AOP-Wiki for any new AOPs that may be pertinent to the subject matter of this paper. Finally, we assessed the frequency of the KEs in the retrievable AOPs. The AOP-Wiki review was conducted on December 13, 2023, and further efforts were based on those findings. The collected AOPs were evaluated for the presence of shared KEs, resulting in a unified AON connecting endocrine disruption to DNT.

### Methodology for Systematic Review

The purpose of a systematic review is to gather and organize existing knowledge on a defined topic. This type of review is distinguished by an unambiguous and replicable methodology.^16^ In our work, we utilized the PRISMA 2020 ^16^ methodology to provide the greatest transparency and informativeness of the results.

Our systematic review aimed to collate all computational models described in the scientific literature that can be employed to predict the occurrence of KEs included in the AON for TD-induced DNT, after exposure to a stressor.

The process of systematic review was divided into four stages and is presented in Figure 1. A detailed description of the process for each stage is provided below.

**Figure 1:**
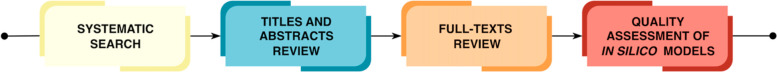
Process of systematic review.

### Methodology of Systematic Search of Scientific Publication

The initial stage involved a systematic search of scientific publications describing predictive models. The methodology employed plays a significant role in determining the quality of the final review results. An inappropriate definition of keywords may result in the identification of numerous irrelevant publications. However, a more significant concern is the omission of essential papers relevant to the investigated topic. To mitigate this risk, considering potential synonyms of keywords in database searches helps ensure comprehensive coverage.

This work aims to gather predictive models with initial KEs in AON as endpoints. Thus, a search string consists of two sets of keywords, with synonyms defined for each. The first one concerned perturbation in thyroxine concentration in serum and neuronal tissues and those defined by Marty et al.^17^ as “non-routine parameters reflecting events of thyroid-related AOPs.” Among these were endpoints not included in the AOP in the AOP-Wiki, but which had been documented in the literature, i.e., inhibition of deiodinases, migration across cell membranes by traveling through specific transporters, and binding to thyroid hormone receptors. The second set of keywords included phrases related to predictive models. The full list of keywords is presented in Table 1.

**Table 1:** Keywords Used for Systematic Search

endpoint		predictive models
endpoint name	keywords for endpoints	keywords for predictive models
decreased thyroxine (T4) in serum	T4 serum OR thyroxine serum	“in silico” “QSAR” “computational model” OR “computational models” OR “computer model” OR “computer models”
	T4 blood OR thyroxine blood	
thyroid hormone levels in neuronal tissue	T4 neuro* OR thyroxine neuro*	
Na^+^/I^–^ symporter inhibition	“Na I symporter” OR NIS	
thyroid peroxidase inhibition	Thyroperoxidase OR TPO	
serum levels of free thyroid hormones	fT4 blood OR free thyroxine blood	
thyroid hormone serum binding proteins	transthyretin OR TTR	
activation, Pregnane-X receptor, NR1l2	“Pregnane X” OR Pregnane-X OR PXR	
inhibition of deiodinases	iodothyronine deiodinase OR DIO OR DIO1 OR DIO2 OR DIO3	
cell membrane transporters	organic anion transporter* AND thyroid	
	L-type amino acid AND thyroid*	
	monocarboxylate AND thyroid*	
	sodium taurocholate polypeptide AND thyroid*	
binding to thyroid hormone receptor	thyroid hormone receptor* AND neuro*	

Search strings were obtained by combining the “endpoint” keyword with the “predictive model” keyword using the Boolean operator “AND” operator. For example, the following search strings were created for the search paper dealing with decreased thyroxine (T4) in serum:

- (T4 serum OR thyroxine serum) AND “in silico”
- (T4 serum OR thyroxine serum) AND “QSAR”
- (T4 serum OR thyroxine serum) AND (“computational model” OR “computational models” OR “computer model” OR “computer models”)
- (T4 blood OR thyroxine blood) AND “in silico”
- (T4 blood OR thyroxine blood) AND “QSAR”
- (T4 blood OR thyroxine blood) AND (“computational model” OR “computational models” OR “computer model” OR “computer models”)

The term “QSAR” was used instead of “(Q)­SAR” in the search strategy, as it is the dominant terminology in bibliographic databases; studies using the “(Q)­SAR” notation are typically indexed under broader in silico descriptors, which were also included.

The full list of all search strings is presented in the Supporting Information (Sheet S1). These search strings were utilized to search two of the most widely used databases for scientific papers: PubMed and Web of Science, on the third of November 20, 2025. Filters were applied to include only peer-reviewed studies in English. Preprints, conference abstracts, and gray literature were excluded to ensure data quality and reproducibility. A concise summary of the search parameters (databases, fields, logic, and filters) is provided in the methodology part, while the complete search strings are listed in the Supporting Information. The workflow of the systematic search is presented in Figure 2.

**Figure 2:**
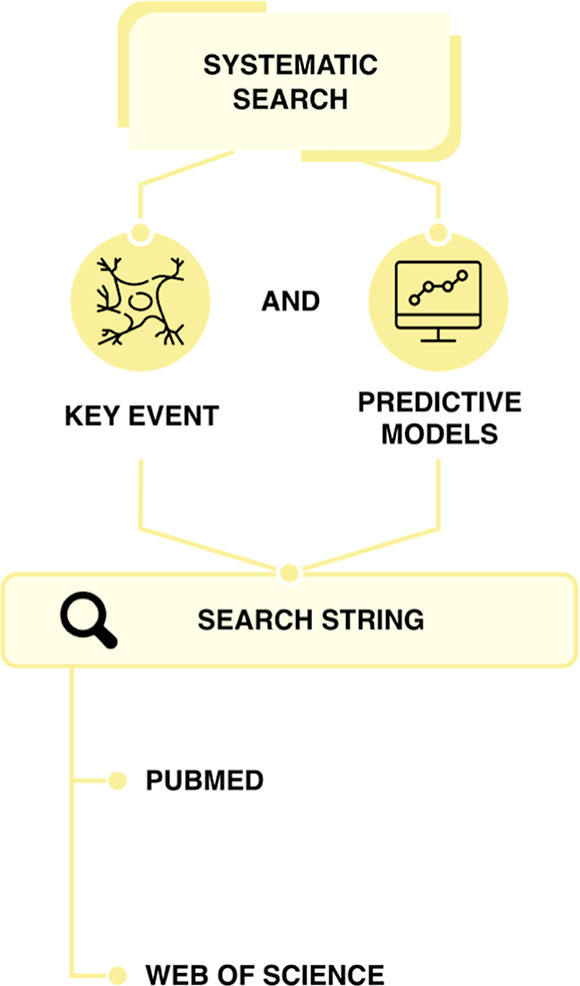
Workflow of systematic search.

### Methodology of the Titles and Abstracts Phase

After the search results based on defined keywords were retrieved, any duplicate publications were removed. Subsequently, the title and abstract of each document were read by two independent reviewers and evaluated. Three possible courses of action exist regarding a reviewed publication: (i) it may be included for further review, (ii) excluded, or, in the case of ambiguity, (iii) marked as “maybe”. The criteria for inclusion and exclusion are presented in Table 2. This phase of the review was conducted using Rayyan, a tool designed to facilitate the systematic review process.^18^

**Table 2:** Inclusion and Exclusion Criteria for the Titles and Abstracts Phase of the Review

inclusion criteria	exclusion criteria
key event(s) in AON as an endpoint(s)	irrelevant topic
QSAR model reported	wrong endpoint
	NON-QSAR predictive model
	non-English documents
	preprints/conference abstracts/gray literature

Upon the completion of all publications assessments by both reviewers, the blind mode in Rayyan was deactivated, revealing each of the reviewers’ decisions. This feature plays a key role in promoting rigorous and unbiased evaluation by hiding the decisions of other reviewers throughout the screening process, thereby preventing cognitive bias and ensuring objective judgment. Once the blind mode was disabled, the reviewers could identify any discrepancies or conflicting decisions. These conflicts were resolved through structured discussion and consensus to ensure consistency and reliability in the selection process. All studies that met the predefined inclusion criteria were subsequently included for further systematic review and detailed analysis.

### Methodology of Full-Texts Phase

The publications extracted in the preceding stages were distributed among the five reviewers. Their objective was to collate data on QSAR models to predict KEs in the AON. The following information was gathered:

- About toxicological data: triggered KE, endpoint (e.g., IC_50_ of TPO), class of chemicals, data source
- About descriptors that characterize stressors: class of descriptors, descriptors used for the prediction, total number of descriptors, software used for prediction, optimization method
- About models: model type (quantitative or qualitative), applied algorithm (e.g., linear regression), and model statistics.

For the purpose of this review, “qualitative” models were defined as classification approaches assigning chemicals to discrete categories (e.g., active/inactive, binder/nonbinder), typically returning a class label and/or probability. “Quantitative” models were defined as regression approaches predicting continuous endpoints (e.g., IC50, *K*_i_, binding affinity, or other potency-related measures). When continuous measurements were ultimately dichotomized by using an activity threshold, the model was classified as qualitative, reflecting its intended decision-making use.

### Methodology of the Quality Assessment of Predictive Models

The deployment of in silico models for predicting the toxicity of chemicals requires the achievement of specific quality standards that indicate reliability. The criteria that a model must fulfill are delineated in the (Q)­SAR Assessment Framework: Guidance for the Regulatory Assessment of (Quantitative) Structure–Activity Relationship Models, Predictions, and Results Based on Multiple Predictions, issued by OECD.^19^ In accordance with the criteria outlined in this document, the evaluated aspects are presented in Table 3.

**Table 3:** Components of Model Evaluation

no.		fulfilled	not fulfilled
1.	Defined Endpoint		
	1.1. clear scientific and regulatory purpose	the rationale for the significance of the research is clearly defined	no explanation of the relevance of the research
	1.2. transparency of the underlying experimental data	data source is specified, e.g., database or paper citation	data source is not specified
	1.3. quality of the underlying experimental data	the authors provided the experimental conditions, or protocols that were employed during the study	conditions of the experiment or the protocol used are unmentioned
2.	Unambiguous Algorithm		
	2.1. description of the algorithm and/or software	the methodology underlying the computer model and software for calculating descriptors is delineated	methodology and/or software are not specified
	2.2. inputs and other options	the publication includes information to identify compounds, e.g., SMILES and software settings	a complete list of compounds and/or software settings was not listed
	2.3. model accessibility	the necessary information is available to reproduce the model. This may include the following: QMRF (QSAR model reporting format) model equation (if possible) complete methodology script or link to a software with a model applied	the information in the manuscript does not allow the model to be reproduces (especially when more complex algorithms such as random forests or neural networks have been used)
3.	A Defined Domain of Applicability (AD)		
	3.1. clear definition of the applicability domain and limitations of the model	the reliability of the predictions for a given substance can be assessed based on the applicability domain of the model, description of AD assessment is required	no or unclear applicability domain
4.	Appropriate Measures of Goodness-of-Fit, Robustness, and Predictivity		
	4.1. goodness-of-fit, robustness	information on the size of the test and training set and the statistic of the model is provided	no information about the size of the test and training set and/or statistics
	4.2. predictivity	statistics about predictivity is provided	no predictivity statistics are provided (e.g., no test-set and/or cross-validation performance)
5.	Mechanistic Interpretation		
	5.1. plausibility of the mechanistic interpretation	the authors provide an indication of the relationship between the given structure elements or descriptors and the mechanism of action	no mechanistic interpretation

The OECD (Q)­SAR Assessment Framework criteria were applied using predefined decision rules to ensure consistent evaluation across the models. A criterion was considered fulfilled when the required information was available either in the main manuscript, the Supporting Information, or through clearly referenced and publicly accessible external resources (e.g., GitHub, Zenodo, or web-based tools). If the information was stated to exist but was not accessible or if critical details required for model identification or execution were missing, the criterion was marked as not fulfilled.

In this review, model accessibility was interpreted as the practical ability to generate predictions for new chemicals, in line with OECD guidance, and was explicitly distinguished from full independent reproducibility. Accordingly, models implemented in commercial software or requiring specialized tools were considered to be accessible when sufficient methodological detail was provided to enable their use by third parties with access to the relevant software. Conversely, models relying on complex workflows, such as those based on 3D descriptors or structure optimization, were considered not accessible when essential steps (e.g., conformer generation, energy minimization, and descriptor calculation settings) were not sufficiently documented to allow execution or reuse. Criteria related to goodness-of-fit and robustness captured training-set characteristics and internal validation approaches (e.g., cross-validation or internal training/test splits), reflecting model stability during development. In contrast, predictivity refers to model performance evaluated on external or explicitly defined predictive validation sets. When reported, the data-splitting strategy (e.g., random, scaffold-based, or temporal split) was recorded as part of the model assessment. This distinction was adopted to clearly separate internal model robustness from evidence of predictive performance.

## Results and Discussions

This study focuses on KEs that describe disorders of the thyroid system and their potential to interfere with the steady development of the nervous system. A comprehensive understanding of the relationship between the structure of chemical compounds and their potential impact on thyroid hormone concentrations may enable the identification of substances that may pose a risk to children, particularly those who have yet to be born. This work summarizes predictive models available in the literature referring to the described relationship based on the AON linking TD with DNT.

### AON for DNT Caused by TD

We initially identified and followed the most recent review gathered, AOP describing the mechanism of inducing DNT via TD provided by Marty et al.^17^ In this article, five AOPs available in the AOP- Wiki were described:

- AOP 8: “Upregulation of Thyroid Hormone Catabolism via Activation of Hepatic Nuclear Receptors, and Subsequent Adverse Neurodevelopmental Outcomes in Mammals”;^20^
- AOP 42: “Inhibition of Thyroperoxidase and Subsequent Adverse Neurodevelopmental Outcomes in Mammals”;^21^
- AOP 54: “Inhibition of Na^+^/I^–^ symporter (NIS) leads to learning and memory impairment”;^22^
- AOP 134: “Sodium Iodide Symporter (NIS) Inhibition and Subsequent Adverse Neurodevelopmental Outcomes in Mammals”;^23^
- AOP 152: “Interference with thyroid serum binding protein transthyretin and subsequent adverse human neurodevelopmental toxicity”.^24^

Furthermore, the AOP-Wiki was reviewed using the keywords delineated in the “Methodology” section, to ensure the most recent results. An additional relevant mechanism was identified: AOP 65: “XX Inhibition of Sodium Iodide Symporter and Subsequent Adverse Neurodevelopmental Outcomes in Mammals”.^25^

Moreover, three further AOPs were identified that did not involve human toxicity: AOP 364: “Thyroperoxidase inhibition leading to altered visual function via decreased eye size”,^26^ AOP 365: “Thyroperoxidase inhibition leading to altered visual function via altered photoreceptor patterning”^27^ and AOP 402: “Thyroid peroxidase (TPO) inhibition leads to periventricular heterotopia formation in the developing rat brain”.^28^ They were not included in the AON.

All six relevant AOPs (AOP 8, 42, 54, 134, 152, and 65) were used to construct the AON, illustrated in Figure 4.

**Figure 3:**
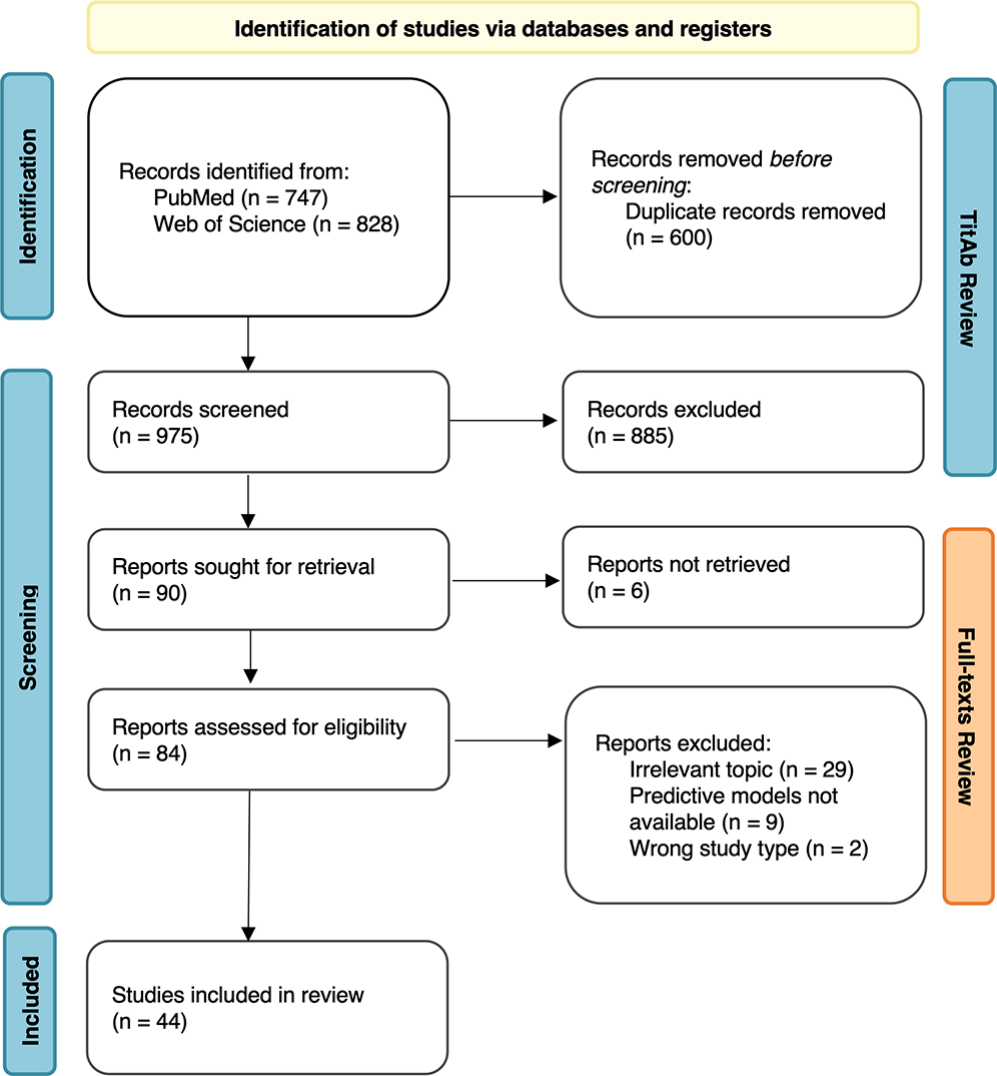
PRISMA 2020 flow diagram.

**Figure 4:**
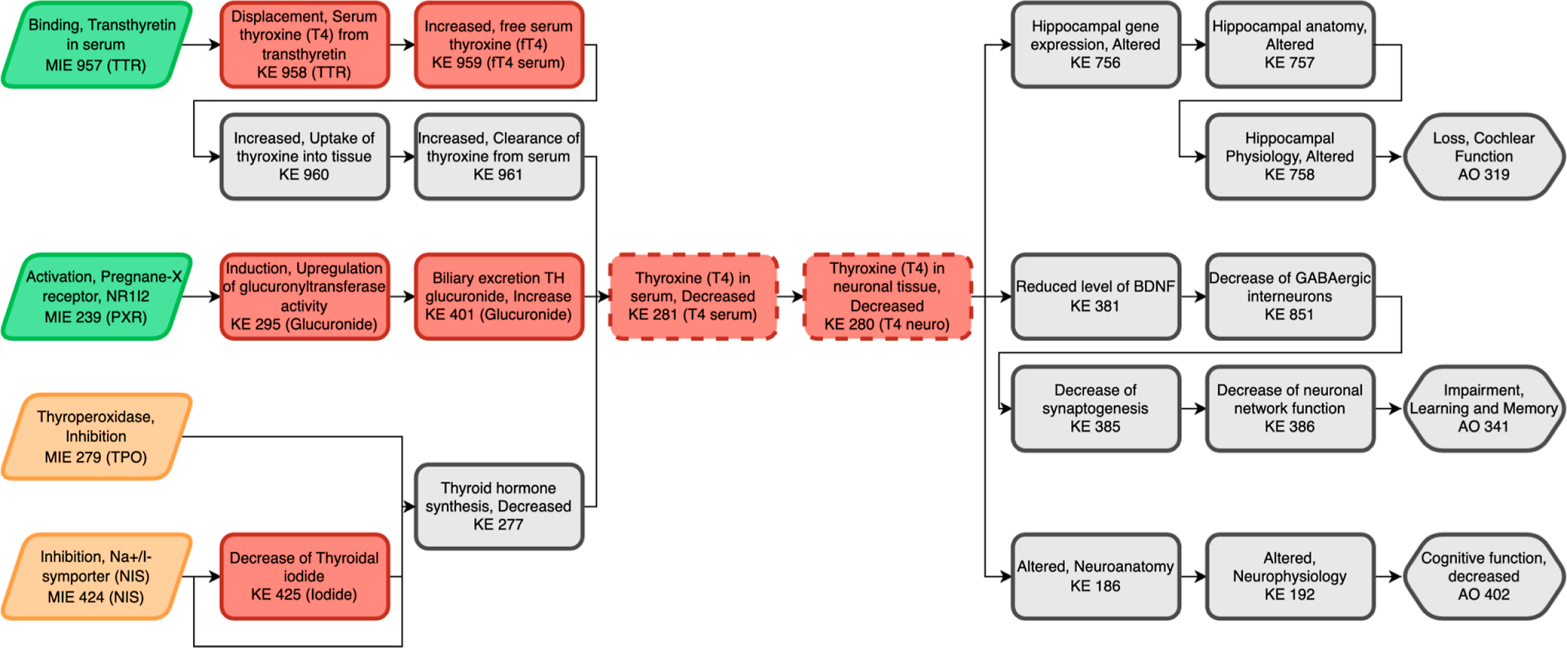
AO Pathway Network for DNT induced by TD. The interpretation of shapes: parallelograms—molecular initiation events, rectangles—KEs, hexagons—AOs. The dashed line represents events that are common to all AOPs. The colors of the boxes indicate the availability and quality of the in silico models. Green denotes that models meeting all quality criteria are available. Orange indicates that models are available, but none meet all quality criteria. Red signifies the absence of models. A literature review was not conducted for KEs in gray boxes.

The KEs are presented as a causal connection, as in the AOP: from left to right. The MIEs are indicated in green, the KEs in yellow and orange, and the AO in red. The entire cascade of KEs commences with four MIEs, which, in turn, lead to two common, consecutive KEs (orange): KE 281: “Thyroxine (T4) in serum, Decreased”^29^ and KE 280: “Thyroxine (T4) in neuronal tissue, Decreased”.^30^ The network then diverges into three pathways that describe developmental malformations of the nervous system. The first pathway concerns changes in the anatomy of the hippocampus, which results in an AO described as a loss of cochlear function (KE 319). The second pathway indicates a decrease in neuronal network function, which leads to impairment of learning and memory (KE 402). The third line depiction is short on details compared with previous ones. It is described only as alterations in neural anatomy and neurophysiology, which result in decreased cognitive function.

Marty et al^17^. identified three events that influence the local regulation of thyroid hormone levels and may contribute to DNT:

- Inhibition of deiodinases,
- Altered thyroid hormone transport (called: Cell membrane transporters),
- Thyroid hormone receptor binding and transcriptional regulation.

However, the complete mechanism relevant for humans incorporating these events has yet to be implemented in the AOP-Wiki, and thus, they are not included in the AON. Nevertheless, as they were identified as nonroutine parameters reflecting events associated with thyroid-related AOPs, they were also incorporated into the literature review.

In conclusion, the work of Marty et al. was used to collect and extend the event framework triggering endocrine-disruption-induced DNT, whereas the primary goal of this review is to assess the availability, quality, and applicability of QSAR models for those events. Therefore, it should be emphasized that this work is complementary in nature and adds value to the review by Marty et al.

### Systematic Review

The objective of the subsequent stage of our research was to conduct a systematic review of the accessible literature related to predictive models for the identified KE. The review process was conducted following the PRISMA 2020 standards. Figure 3 presents a flowchart summarizing the number of publications reviewed at each stage. A detailed statistical data, with a particular focus on each KE, can be found in the Supporting Information (Sheet S1).

### Title and Abstracts Phase

The systematic review followed a rigorous process of identifying, screening, and including relevant studies to ensure the highest levels of quality and relevance. The systematic review commenced with an extensive search across two key scientific databases, PubMed and Web of Science, which collectively yielded 1575 records (747 from PubMed and 828 from Web of Science). In the initial phase, duplicates were identified and removed, resulting in the exclusion of 600 duplicate entries, leaving 975 unique records for further assessment. These 975 records were then screened based on predefined inclusion criteria, Table 2, which focused on the study’s relevance to the review’s objectives and scope. During this screening, 885 records were excluded, as they did not align with the inclusion criteria pointed out, leaving 90 publications identified as potentially relevant for further review. The most common reason for exclusion was the irrelevance of the topic. Many articles searched for MIEs involved molecular dynamics and molecular docking. Although this methodology relies on prediction, it does not include the quantitative relationships between structure and activity; thus, we excluded them from further steps. On the other hand, the papers on early KEs were reviews that described mechanisms or qualitative relationships. Studies on zebrafish were also identified, and they are considered as a model organism for DNT. The 90 papers marked as relevant were subsequently sought for retrieval, marking the transition from the title and abstract screening phase to the more detailed full-text evaluation phase.

### Full Texts Phase

Out of 90 potentially relevant publications, six could not be retrieved due to inaccessibility, reducing the number of studies eligible for full-text assessment to 84. In this phase, a more rigorous and detailed review of the full text was conducted to evaluate the studies against all predefined eligibility criteria from Table 2. During this process, 40 publications were excluded: 29 were deemed irrelevant to the specific topic of the review, 9 were excluded due to the unavailability of predictive models, and 2 were excluded for being a conference material instead of a scientific paper. After these exclusions, 44 studies were found to fully meet the eligibility criteria and were included in the final systematic review. This process ensured a high level of scrutiny and rigor, as the review team carefully evaluated each study’s relevance, methodological quality, and contribution to the overall research objectives. By following these meticulous steps, the systematic review was able to include only the most relevant and scientifically robust publications, enhancing the credibility and reliability of the conclusions.

A comprehensive overview of the number and types of models found for various biological endpoints, categorized into qualitative and quantitative approaches, is provided in Table 4. The endpoints evaluated include MIE 239 (acronym: PXR), MIE 279 (acronym: TPO), MIE 424 (acronym: NIS), and MIE 957 (acronym: TTR), which are part of the Adverse Outcome Pathway Network (AOPN). In addition, predictive models were identified for other key events (KEs), such as the inhibition of deiodinases (acronym: DIO), cell membrane transporters (acronym: Membrane), and thyroid hormone receptors binding (acronym: THR). Although 44 publications were screened, many of these studies utilized more than one predictive model, resulting in the total number of models equal to 178. This reflects the diverse application of multiple modeling approaches across considered endpoints. Full details of the models (i) endpoint and descriptors: KE, defined endpoint, class and number of chemicals used to model development, organism, data source, class, number, type, and software regarding the descriptors; (ii) methodology: model type and method, available statistics, including their specific use cases, are provided in the Supporting Information (endpoint, class and number of chemicals used to model development, organism, data source, class, number, type, and software regarding the descriptors; (ii) methodology: model type and method, available statistics, including their specific use cases, are provided in the Supporting Information (Sheet S2). Sheet S2 in the Supporting Information also contains the elements of the model assessment based on OECD principles (QSAR Assessment Framework (QAF) documentation^31^), for which results are described in the next subchapter.

Importantly, the qualitative-predictive splits reflect two distinct use cases and data constraints. Qualitative models are typically aligned with hazard identification and prioritization, where a categorical decision is sufficient and heterogeneous assay outputs can be harmonized via activity thresholds. Quantitative models aim to predict potency and therefore require substantially higher endpoint consistency and reporting detail, which limits their availability and cross-study interpretability. In practice, the distribution observed in Table 4 suggests that the current literature is stronger in screening-level prediction than in robust potency estimation.

**Table 4:** Statistics of Predictive Models That Are Available in the Literature

		molecular initiating events and key events (KEs)						
	models	PXR	TPO	NIS	TTR	DIO	membrane	THR
qualitative	Bayesian classifier	20	0	0	0	0	0	0
	consensus	1	3	0	0	0	0	0
	decision tree	3	0	0	6	0	0	0
	extreme gradient boosting	0	3	1	0	3	0	0
	k-nearest neighbor	5	1	0	13	0	0	0
	logistic regression	1	2	1	0	3	0	0
	neural networks	4	2	1	1	3	0	0
	random forest	5	3	1	1	3	6	0
	support vector machine	9	2	1	1	3	6	0
	other	3	3	0	5	0	0	0
quantitative	heuristic method	9	0	0	0	0	0	0
	k-nearest neighbor	0	1	0	0	0	0	1
	multiple linear regression	4	0	0	10	0	0	1
	partial least squares	2	0	0	7	0	0	0
	other	6	1	0	1	0	0	6
SUM		72	21	5	45	15	12	8

The development of predictive models is grounded on experimental data. It can be observed that the higher level of biological organization relating to a KE is associated with an increase in the complexity, time, and cost consumption of assessing the occurrence of that KE. For instance, the effect of exposure to a substance on serum thyroxine levels necessitates in vivo testing (see Marty et al. for recommended guidelines). Meanwhile, for MIEs, experiments can be conducted using cell lines and even employ molecular dynamics. This yields a greater abundance of data elucidating receptor interactions than substances’ effects on tissues, organs, or the entire organism. We postulate that this may be the underlying reason for the scarcity of available models for KEs other than MIEs.

Endpoints like PXR and TTR receive the most attention, with 72 and 45 models, respectively, demonstrating the priority placed on pathways related to metabolism, detoxification, and hormone transport.^32–34^ PXR models utilize a wide variety of predictive models, including Bayesian classifiers, Random Forest, and Support Vector Machines (SVMs), alongside quantitative methods, such as heuristic models and linear regression. These approaches reflect the need to predict the interaction of chemicals like food additives, plasticizers, flame retardants, and pollutants (e.g., PFAS and PCBs) across different exposure scenarios. Similarly, TTR models, using k-Nearest Neighbor, Random Forest, and regression techniques, focus on substances like PFAS, brominated flame retardants (BFR), and phenolic byproducts, which are known for their persistence and endocrine-disrupting potential.

The large body of PXR models likely reflects the broader role of PXR as a xenobiotic sensor widely studied in drug metabolism and metabolic disease context; consequently, not all retrieved PXR-focused studies were originally framed as endocrine disruption research, yet they remain informative for this MIE within the thyroid-disruption-driven AOP network.

In contrast, NIS and THR are significantly underrepresented, with only 5 and 8 models, respectively. This limited coverage reflects the challenges of modeling pathways such as iodine uptake and receptor binding, which demand detailed biological data and an intricate mechanistic understanding. NIS models utilize predictive approaches, including Decision Trees, SVMs, and Neural Networks. While Neural Networks provide advanced modeling power by capturing nonlinear relationships, their reliance on large data sets and intensive computational resources may contribute to their limited use in this endpoint. Instead of simply aiming to increase the quantity or complexity of models, a strategic focus on developing innovative and diverse modeling approaches is needed to expand the domain and adapt it to new areas of research or chemical classes. This would facilitate broader exploration and enhance the relevance of these models in untapped fields. Similarly, THR models, although focused on receptor-binding ligands critical to thyroid regulation, remain sparse, with only a few heuristic models available. This endpoint’s minimal coverage highlights the need for more advanced predictive models capable of better capturing receptor interactions and their role in hormonal balance. Both NIS and THR illustrate key gaps in the predictive model network, underscoring the importance of innovative developments in these areas to support comprehensive chemical risk assessment.

Although there are 21 models associated with TPO primarily focusing on chemicals found in agriculture, cosmetics, and pharmaceuticals, they rely mostly on heuristic methods. Similarly, DIO models, comprising 15 entries, explore the inhibition of deiodinase enzymes with an emphasis on pharmaceuticals, but the use of Random Forest and SVMs highlights a need for more diverse and advanced predictive models.

The Membrane Transporter endpoint, with 12 models, emphasizes a mode profound gap. The relevant chemical classes remain undefined, pointing to the need for additional research to explore the transport mechanisms affecting thyroid hormone regulation. While qualitative predictive models like Random Forest and SVM dominate, the lack of clearly characterized chemical entities suggests insufficient experimental data.

In addition, an analysis of the relationship between chemical classes and reported predictive models was provided. There are nine categories of chemical classes for which QSAR models were developed (Table 5). The dominant category (143) is the “Other” which means that most of the reported models were built on data sets containing undefined chemical classes. Excluding the “Other” category, PFAS is the most represented specific class with 11 models. This reflects the high current research interest in “forever chemicals”. BFR and PDE groups follow with 6 and 5 models, respectively, while classes such as halogenated thiophenols, PCBs, and PH have the fewest dedicated models in this data set, ranging from 1 to 2. The data clearly indicate a significant imbalance in the current modeling landscape. While there is a vast number of general models (categorized as “Other”), the specific classes, many of which are high-priority environmental pollutants or bioactive substances, have relatively few dedicated models.

**Table 5:** Distribution of Chemical Classes

chemical class	brominated flame retardants (BFR)	cosmetic ingredients (CI)	halogenated thiophenols (HTP)	polychlorinated biphenyls (PCB)	polybrominated diphenyl ethers (PDE)	perfluorinated and polyfluoroalkyl substances (PFAS)	(PFAS/BFR)	steroids (ST)	other
no. of models	6	5	1	2	5	11	2	3	143

### Quality of Predictive Models

Following model collection, their quality was evaluated individually based on the criteria in Table 3. The complete results for all 178 models can be found in the Supporting Information (Sheet S2). Furthermore, a graphical representation of the results is presented in Figure 4 as the availability or absence of good-quality models plotted on the AON. For two KEs (green color): TTR and PXR 18 and 14 models, respectively, meet all of the quality criteria. The remaining five KEs require further clarification of one or more aspects to comply with the criteria set forth by the OECD. The issue that was identified most frequently was model availability (120 instances), followed by an unclearly defined applicability domain (78 instances). The authors omitted a mechanistic interpretation of the models with a comparable frequency (72 cases).

In addition, the model validation strategies and performance reporting were heterogeneous across the identified studies. Many models relied primarily on internal validation (e.g., random train/test splits and cross-validation), whereas external validation on truly independent chemical sets was less frequently reported. Furthermore, the reported performance metrics varied by model type: qualitative (classification) models typically provided measures such as accuracy, sensitivity/specificity, or AUC, while quantitative (regression) models reported metrics such as R^2^, RMSE, and/or MAE. In several cases, the separation between training performance and predictive performance (test set or cross-validation) was not clearly stated, which can lead to optimistic estimates of real-world predictivity. For transparency, we therefore summarize the validation approach (internal vs external) and the available predictive metrics for each model in the Supporting Information (Table S2).

The majority of models (120) fail to meet criterion 2.3, which concerns model accessibility, an important criterion that clearly indicates which models can be used for predictions. For simple regression models, it is only necessary to specify the equation, after which the endpoint value can be calculated by substituting the descriptor values. However, reproducing an identical model may not be possible for more complex models with sophisticated algorithms such as Random Forest or Neural Networks, despite a thorough description of the methodology.

Addressing these gaps requires a more integrated and strategic approach. Developing Key Event Relationships (KERs)—causal links between KEs—can create a more interconnected model network, reducing redundancy and enhancing the interpretability of chemical interactions. Leveraging existing mechanistic insights in this way aligns with regulatory priorities, helping to improve predictive models without unnecessary proliferation.

A more comprehensive research focus is essential, emphasizing advanced in silico methods and a deeper integration of mechanistic insights. Expanding model coverage for underrepresented endpoints and establishing robust KERs will further enhance the predictive power and clarity of these models. Refining existing models and embracing computational advancements will allow the research community to build reliable frameworks for chemical safety assessments. These efforts align with the principles of reduce, refine, and replace (3Rs) in toxicology, minimizing reliance on animal testing and fostering the development of NAMs for efficient, ethical, and comprehensive risk assessments.

The AOP-anchored mapping of in silico models presented in this review provides a practical starting point for their integration into existing regulatory frameworks, such as REACH, the Biocidal Products Regulation, or US EPA NAMs. In a screening context, QSAR models linked to early MIEs (e.g., PXR activation, TTR binding) may support rapid prioritization of substances for further assessment. For chemicals triggering these early events, follow-up testing using in vitro DNT batteries could be considered to probe downstream neurodevelopmental effects, particularly for thyroid-mediated mechanisms.

Where concordant evidence from in silico and in vitro NAMs indicates potential DNT concern, escalation to higher-tier testing may be warranted, whereas negative or inconclusive results may justify refinement of exposure assessment or targeted data generation. Although the present review does not propose a formal regulatory decision tree, the identification of model availability, quality, and gaps along the TD-DNT AOP provides a transparent basis for developing integrated testing strategies and evidence-based regulatory workflows in future work.

Several limitations of this review should be acknowledged. First, as with any literature-based synthesis, the results are subject to publication bias, as studies reporting successful or well-performing models are more likely to be published than negative or inconclusive findings. In addition, the systematic search was restricted to peer-reviewed articles published in English, which may have excluded relevant studies reported in other languages or regulatory reports. Second, the chemical space covered by the identified models is uneven and shows a clear bias toward well-characterized industrial compounds, reflecting the availability of experimental data rather than the full diversity of environmental chemicals. This skew limits the generalizability of some models, particularly when applied to poorly represented chemical classes. Third, heterogeneity in reported performance metrics and validation strategies complicates direct comparison across models and endpoints. While some studies report external validation or multiple predictivity metrics, others rely primarily on internal cross-validation. Models evaluated exclusively using cross-validation may provide optimistic estimates of performance, and their predictive reliability for new chemical space should therefore be interpreted with caution. Finally, although cross-validation is widely used during model development, overinterpretation of CV-only models without independent validation or a clearly defined applicability domain may lead to misplaced confidence in regulatory contexts. These limitations underscore the need for improved data sharing, harmonized reporting practices, and greater emphasis on external validation when developing in silico NAMs for DNT assessment.

## Conclusions

This systematic review summarizes the current state of knowledge on in silico approaches employed so far for the assessment of TD-induced DNT. The mechanisms responsible for this type of toxicity have been adapted using the 6 AOPs available in the AOP-Wiki database. However, as neurotoxicology continues to advance, new mechanisms will be developed.^35^

In view of the 3Rs of animal research and the future update of Annex II, the development of NAMs to assess TD-induced DNTs is important.^7^ Collaboration between laboratory- and computational-based method scientists is essential in the implementation of new, efficient tests that will enhance the safety of the chemicals.

The existing computational literature primarily concentrates on the events that initiate the cascade of effects leading to the DNT. The development of an advanced battery of in silico tests to predict the probability of triggering KEs on the AON would represent a significant scientific advance. However, to achieve this, it is necessary to develop predictive models of the remaining KEs. Furthermore, it is essential that the quality of the models utilized is high, ensuring that the outcomes are indisputable. Attainment of the “model accessibility” criterion in QAF facilitates the replication and utilization of the model. A clearly defined applicability domain allows for a straightforward assessment of the reliability of predicted outcomes for the substance under examination. Moreover, mechanistic analysis enables the distinction between a random correlation between descriptor and endpoint and the possibility that the structural features in question are indeed responsible for the induced effect.

A key constraint for improving and extending in silico coverage is the experimental data gap, particularly for downstream KEs at higher levels of biological organization. Limited availability of well-annotated data sets, heterogeneous assay conditions, and scarce independent test sets restrict both model development and robust external validation, which in turn slows progress beyond MIE-focused predictions. Addressing this bottleneck will require targeted data generation for underrepresented KEs and more systematic FAIR (Findable, Accessible, Interoperable, Reusable) reporting and sharing of experimental outcomes to enable reproducible model training and benchmarking.

In summary, our analysis of 44 identified studies and 178 individual models reveal a significant concentration of research efforts on specific endpoints, most notably PXR (72 models), TTR (45), and TPO (21). However, the high quantity of available tools does not necessarily translate to regulatory readiness. The fact that only 32 models (approximately 18%) achieved full compliance with the OECD QAF criteria serves as a critical benchmark for the field. These findings quantitatively demonstrate that future research must shift its focus from broad model generation toward the refinement and rigorous validation of endpoint-specific tools to ensure they meet the stringent requirements of modern risk assessment frameworks.

In conclusion, improvements to existing computational models or the development of new ones (applicable to a wider range of DNT-related KEs), are needed to propose new strategies for a more detailed risk assessment. Combining all the gathered knowledge, including in vivo, in vitro, and in silico methods, will it be possible to investigate the mechanisms leading to the disability of the developing nervous system via TD?

## Supplementary Material



## Data Availability

The data supporting this article have been included as part of the Supporting Information.

## Notes

Complete PubMed and Web of Science search strings with record counts, including deduplication and title/abstract screening outcomes; and extracted in silico models identified in the systematic review (model metadata and reported performance), including OECD (Q)­SAR Assessment Framework-based quality assessment for each model (XLSX)

## Abbreviations:

AD: applicability domain
ADHD: attention deficit hyperactivity disorder
ADMET: absorption, distribution, metabolism, excretion, and toxicity
AON: adverse outcome network
AOP: adverse outcome pathway
AO: adverse outcome
ANT: adult neurotoxicity
BFR: brominated flame retardants
BPA: bisphenol A
BPR: biocidal products regulation
CI: cosmetic ingredients
CV: cross-validation
DIO: iodothyronine deiodinase
DNT: developmental neurotoxicity
ECHA: European Chemicals Agency
ED: endocrine disruption/endocrine disruptor
FAIR: findable, accessible, interoperable, reusable
IVB: in vitro battery
KE: key event
KER: key event relationship
MIE: molecular initiating event
NAM: new approach methodology
NIS: sodium/iodide symporter
OECD: Organisation for Economic Co-operation and Development
PBDE: polybrominated diphenyl ethers
PCB: polychlorinated biphenyls
PFAS: per- and polyfluoroalkyl substances
PH: pharmaceuticals
PRISMA: preferred reporting items for systematic reviews and meta-analyses
PXR: Pregnane X receptor
QAF: (Q)­SAR assessment framework
QMRF: QSAR model reporting format
(Q)­SAR: (quantitative) structure–activity relationship
SVM: support vector machine
TD: thyroid disruption
TG: test guideline
TH: thyroid hormone
THR/TR: thyroid hormone receptor
TPO: thyroid peroxidase
TTR: transthyretin
T3: triiodothyronine
T4: thyroxine
